# Validation of the interview-based life-space assessment in institutionalized settings (LSA-IS) for older persons with and without cognitive impairment

**DOI:** 10.1186/s12877-020-01927-8

**Published:** 2020-12-10

**Authors:** Klaus Hauer, Phoebe Ullrich, Patrick Heldmann, Saskia Hummel, Jürgen M. Bauer, Christian Werner

**Affiliations:** 1 AGAPLESION Bethanien Hospital Heidelberg/Geriatric Centre of the University of Heidelberg, Rohrbacher Str. 149, 69126 Heidelberg, Germany; 2 Center of Geriatric Medicine, Heidelberg University, Heidelberg, Germany; 3 Network Aging Research (NAR), Heidelberg University, Heidelberg, Germany; 4 Medical Faculty of the Heidelberg University, Heidelberg, Germany

**Keywords:** Assessment, Clinical trial methods, Cognitive impairment, Life-space mobility, Measurement, Exercise, Physical activity, Validation

## Abstract

**Background:**

Self-reported life-space assessment methods so far focus on community-dwelling persons, with a lack of validated assessment methods for institutionalized settings. This study evaluated construct validity, test-retest reliability, sensitivity to change, and feasibility of a new Life-Space Assessment for Institutionalized Settings (LSA-IS) in geriatric patients.

**Methods:**

Psychometric properties of the LSA-IS in 119 hospitalized geriatric patients (83.0 ± 6.2 years) with and without cognitive impairment (CI) [Mini-Mental State Examination: 22.4 ± 4.9 scores] were evaluated within a comprehensive validation design. For the total group and subgroups according to cognitive status, construct validity was assessed by calculating Spearman’s rank correlation coefficients (rho) with established construct variables, test–retest reliability by intra-class correlation coefficients (ICCs), sensitivity to change by standardized response means (SRMs) calculated for effects of early ward-based rehabilitation during hospital stay.

**Results:**

The LSA-IS (total score) demonstrated good test–retest reliability (ICC = .704), and large sensitivity to change (SRM = .806), while construct validity was small to high indicated by significant correlations of the LSA-IS to construct variables (rho = .208–716), depending on relative construct association. On average results of LSA-IS sub-scores confirmed results of the total score. Subgroups according to cognitive status did not differ for most analyzed variables. A completion rate of 100% and a completion time of 3.2 ± 1.2 min documented excellent feasibility.

**Conclusions:**

The interview-based LSA-IS has proven to be valid, reliable, sensitive, and feasible in hospitalized, multi-morbid, geriatric patients with and without CI documenting good psychometric properties for institutionalized settings.

**Trial registration:**

DRKS00016028

## Background

Mobility in institutionalized settings is severely restricted with the consequence of a highly sedentary behavior of older patients during hospital stay or nursing home residents spending most of their time lying or sitting [1, 2]. This immobility is associated with relevant, negative consequences such as drastic functional decline and muscle loss [3, 4], and higher risk for nursing home placement and mortality following hospitalization [5]. While mobility in community-dwelling older persons is influenced by a large number of individual as well as societal factors [6, 7], in institutionalized persons some additional factors become specifically relevant [8]. Such factors with influence on mobility status during hospitalization cover patient-related (illness severity, comorbid conditions), treatment-related (bed-rest required, hospital devices such as catheters, restraints), attitudinal (attitudes towards mobility, expectations of hospital stay), and institutional aspects (nursing to patient ratio, availability of equipment) [9]. Institutions such as hospitals or nursing homes also have an overwhelming organizational influence by their treatment routines or social activities (e.g. timing/ location of meals) [10].

Among the vulnerable, multi-morbid persons in institutionalized settings, persons with CI stand out, as they show a decreased health status compared to their non-impaired peers [11, 12], relevant for their health prognosis but also for health assessments. To address the specific limitations and resources of this vulnerable group, it is mandatory to specifically develop and validate assessment methods in this population [13–15].

To better understand health trajectories while being institutionalized, a large number of established assessments and diagnostic procedures have been established also including functional status or motor performance. Surprisingly the concept of life-space mobility (LSM) has not made it yet into these diagnostic routines despite its’ unique ability to cover mobility-related quality of life and its’ potential role as a “biomarker” of health status, documenting the individual habitual physical activity range in contrast to highly standardized testing routines. For assessment purposes the LSM concept offers chances as well as challenges as it is determined by various domains related to the individual status as well as social interaction to others [7, 16].

A number of life-space assessment methods have been developed and validated for use in community dwelling persons such as the “Life-Space Diary” [17], the “Life-Space Questionnaire” [18], the “University of Alabama at Birmingham – Life-Space Assessment” (UAB-LSA) [16], the “Life-Space Assessment in Persons with cognitive impairment” (LSA-CI) [14], and the “Life-Space mobility at home assessment” [19]. However, different validation approaches have been used for these life-space measures. Construct validity was analyzed in all validation studies, but the comprehensiveness of constructs used differed substantially. Only one study selected variables based on a comprehensive theoretical, multi-domain framework on mobility [14], while literature-based constructs including variables of two or three domains as identified in previous comparable research [16, 18] or measures with strict focus on one domain (physical function) [17, 19] have been used in other studies. Reliability was tested in terms of intra-rater reliability [19], and test-retest reliability [14, 16, 18]. Feasibility was analyzed in only few studies, with one study presenting a comprehensive documentation of duration of assessment, completeness of reports, and floor/ceiling effects [14] and other studies reporting on potential ceiling effects [16] or completeness [17]. Sensitivity to change has been the least analyzed psychometric property performed in two studies [14, 16].

The only LSM assessment (The “Nursing Home Life-Space Diameter”, NHLSD) which could be identified for an institutionalized setting [20], performed a validation with reliability and restricted construct validity with functional-social variables, but not an evaluation of comprehensive construct validity, feasibility, or sensitivity to change. Level of personal support represents an option for data assessment but was not included in presented data of the validation. The NHSLD was designed to be nurse-administered which may differ from a patient perspective with additional burden to nurses. As with other established mobility assessment methods, the evaluation tool was not specifically tailored and validated for use in multi-morbid, vulnerable populations such as persons with cognitive impairment (CI), with relevant potential limitations for self-report, although such vulnerable persons represent the majority in a large number of institutionalized settings. The fixed time frame of 2 weeks of the NHLSD is not feasible at least in hospital settings with varying duration of hospital stay, most often less than 2 weeks.

The objective of the present study therefore was to comprehensively validate a new, detailed, interview-based LSM assessment, specifically developed for institutionalized persons with and without CI including construct validity, test-retest reliability, sensitivity to change, and feasibility in hospitalized geriatric patients including subsamples according to cognitive status.

## Methods

### Study design

The present study follows a comprehensive validation design (see Additional file 3) using secondary data from a prospective, longitudinal cohort study examining physical activity behaviour and mobility of geriatric patients with and without CI during hospitalization (trial registration number: DRKS00016028). The study was performed according to the Helsinki Declaration and was approved by the ethics committee of the Medical Department of the University of Heidelberg (S-709/2018).

### Study sample

Participants were consecutively recruited from acute medical wards of a geriatric hospital. Individuals receiving complex early geriatric rehabilitation treatment according to the German hospital payment system (German Diagnosis-Related Groups) were included by the following inclusion criteria: age ≥ 65 years, Mini-Mental State Examination (MMSE [21]) score ≥ 10, ability to walk at least 4 m with or without walking aid, no terminal illness, no severe functional, sensorial or behavioral restrictions, which did not allow study participation or assessment, no delirium (Confusion Assessment Method [22]), no uncontrolled infection, adequate language level, and written informed consent (obtained from patients or their legal representatives) within 72 h after admission.

### Descriptive measures

Descriptive data were collected at baseline from patient charts after hospital admission to describe the sample and to analyze construct validity. Trained interviewers assessed measures for health status (weight and height to calculate Body Mass Index (BMI), number of medication, frailty status (Clinical Frailty Scale, CFS [23], falls in the previous year [24], pain (Present Pain Intensity scale, 6 points, PPI [25]), cognitive status (MMSE), and psychological status (health-related quality of life (EuroQol-questionnaire, EQ-5D-3L [26]), apathetic symptoms (Apathy Evaluation Scale- Clinical version, AES-C [27]), and concerns about falling (Short Falls Efficacy Scale-International, 7-item version, Short-FES-I [28, 29]). Motor–functional status was assessed by the Activities of Daily Living (ADL, Barthel-Index [30]), the Short Physical Performance Battery (SPPB [31]), and gait speed from the SPPB. Physical activity (reported as number of steps) was assessed for a 48 h period using the uSense sensor, which has been validated in multi-morbid, geriatric patients [32].

### Clinical intervention

The “complex early geriatric rehabilitation” as analyzed in the present study represents an established rehabilitation routine/pathway to prevent loss of function during in-hospital, acute medical treatment. It was developed as a multidisciplinary, geriatric rehabilitation program including at least two of the following four domains: physiotherapy, occupational therapy, speech and language therapy, and psychology (Operation and Procedure Code version 2018, German Institute of Medical Documentation and Information), (https://www.dimdi.de/static/de/klassifikationen/ops/kode-suche/opshtml2020/).

### Life-space assessment

The Life-Space Assessment in Institutionalized Settings (LSA-IS) is a newly developed, interview-based instrument tailored for the specific target setting to assess LSM by the spatial extent of movement, the frequency of movement, and the level of assistance for movement within institutions during the previous day. The short time frame allows to evaluate trajectories of LSM in institutionalized settings, considering the special needs of persons with acute or chronic medical events and/or CI as well as institution−/organization-based restrictions in LSM. The LSA-IS’ basic structure is defined by life-space zones within a typical institutionalized setting ranging from a person’s own room to outside the facility (e.g. garden of nursing home and beyond the outdoor area of the facility (e.g., public area, neighborhood) to document also higher functioning mobility levels (outdoor), which are still preserved in a minority of institutionalized persons.

The LSA-IS also documents the independence of mobility (i.e., without any support, with equipment or personal support) by a detailed qualitative and quantitative approach. Such a semi-qualitative approach seems mandatory given the low functional status, the extraordinary high risk of falling, and the high implementation level of equipment-, or staff- supported mobility in institutionalized settings such as geriatric hospitals or nursing homes.

The interview procedure of the LSA-IS includes an interview-based and strictly standardized face-to-face questionnaire with a clear focus on the subjective self-report of participants rather than proxy reports. Assessment strategies of the LSA-IS were thus optimized to gain correct and complete information including multi-morbid persons with and without CI. The interview is based on an informal conversational approach to prevent fear of failure in comprehension and recall by precise and structured questions and response options, reduction of recall period, clear structure of the observation period by referring to daily routines and landmark events such as meals, doctoral visits, or therapies, or special events such as external visits, and a summary of the given information for immediate review of reports. This approach is based on previous research to validly assess LSM [14] and physical activity for a short recall period (24 h) in older persons with CI [13, 33, 34].

The LSA-IS is structured by three criteria: A) spatial extent of movement, classified into five hierarchically structured, concentric zones (level 1 = own room, level 2 = within the ward, level 3 = within the facility, level 4 = immediate outdoor area of the facility, level 5 = beyond the area of the facility); B) frequency of movement (1 = 1 × per day, 2 = 2–3 × per day, 3 = 4–5 × per day, 4 = > 5 × per day); and C): independence of movement (1 = with personal support, 1.5 = with equipment, 2 = without any support).

A score for each life-space zone is calculated by multiplying values for the zone, frequency and independence. Each life-space zone is added to a LSA-IS-T total score, with the lowest score of 0 indicating total immobility (bed-bound) and the maximum score of 120 indicating independent mobility beyond the area of the facility at least six times at the relevant day. In addition, three LSA sub-scores can be determined for the maximal life-space zone achieved (1) with equipment or personal assistance if needed (LSA-IS-M, range 0–5), (2) with equipment, if needed, but without personal assistance (LSA-IS-E, range 0–5), and (3) independently without any assistance (LSA-IS-I, range 0–5) [14, 16]. For details of the test proceeding see manual attached in supplements (Additional files 1 and 2).

### Assessment of measurement properties

The analysis of construct validity, test-retest reliability, and sensitivity to change was performed for the total group and subgroups according to cognitive status as a most relevant criteria for rehab prognosis also considering the high incidence of CI in institutionalized settings. Study participants with MMSE scores < 24 (range: 10–23) were considered cognitively impaired and participants with scores ≥24 (range: 24–30) cognitively intact [21].

### Construct validity

Assessment of construct validity was conducted after hospital admission. Items for construct validity were selected according to previous, directly comparable validation studies for life-space assessment for the UAB-LSA [16], the LSA-CI [14] and the NHLSD [20], guided by and categorized according to the comprehensive and well-established mobility model by Webber [6]. Correlations between the LSA-IS scores at baseline with demographic variables (age, gender), measures for health status (BMI, number of medication, frailty status (CFS, falls, pain), cognitive status (MMSE), psychological status (EQ-5D, AES-C, FES-I,), motor - functional status (ADL Barthel Index, SPPB, gait speed), and physical activity (assessed by uSense and reported as number of steps, duration of lying, activity, and gait) were calculated.

### Test-retest-reliability

The LSA-IS assessment was conducted on two consecutive days with general assessments being performed by the same trained interviewer to exclude inter-rater variability.

### Sensitivity to change

Sensitivity to change was examined in all participants tested at admission and at the end of the hospital stay immediately before discharge for effects of the complex early geriatric rehabilitation on LSM.

### Feasibility

Completion rate and completion time for the questionnaire were documented at baseline to determine feasibility. In addition, LSA-IS scores at baseline were checked for floor and ceiling effects, which were considered present when more than 15% of the individuals achieve the lowest or highest score [35].

### Statistical analysis

Descriptive data were presented as frequencies and percentages for categorical variables, and means and standard deviations or medians and ranges for continuous variables as appropriate. Comparison with respect to descriptive characteristics between groups according to cognitive status was conducted using *t*-tests and Mann-Whitney-*U* tests as appropriate.

Spearman’s rank-order and point-biserial correlation coefficients between LSA-IS scores and a comprehensive set of associated factors selected in accordance with a theoretical framework and existing research evidence were calculated to assess construct validity. Correlation coefficients (*r*) were interpreted as small (*r* = 0.1–0.3), moderate (*r* = 0.3–0.5), or high (*r* > 0.5) [36].

Independent from formal cut-off criteria, we hypothesized a priori low to moderate associations of the LSA-IS scores with health-related, psychological and cognitive variables and moderate to high correlations with motor-functional variables and variables documenting physical activity behavior.

Intra-class correlation coefficients (ICC) with 95% confidence intervals for the LSA-IS total score and each sub-score were used to analyze test–retest reliability. ICCs were interpreted as poor (< 0.4), fair to good (≥ 0.4 ≤ 0.75), and excellent (> 0.75) [37]. Sensitivity to change was assessed using paired *t*-tests to test for significant within-group differences between assessments at admission and immediately before discharge and standardized response means (SRMs) to quantify the magnitude of changes. SRMs were calculated as the difference in mean change scores divided by the *SD* of the change score [38]. SRMs were adjusted for the size of correlation coefficients between the baseline and post-intervention scores [39] to use Cohen’s thresholds for effect sizes (trivial < 0.2, small ≥0.2 < 0.5, moderate ≥0.5 < 0.8, and large ≥0.8) [36]. The level of significance was set to *p* < 0.05. All statistical analyses were performed using the Software IBM SPSS Statistics Version 23 for Windows (IBM Corp., NY, USA).

## Results

### Participants’ characteristics

The study sample included 119 multi-morbid (10.2 ± 4.3 medications at admission), older (mean age 83.0 ± 6.2 years) persons with CI (MMSE score: 23 (10–30) points) and motor impairment (SPPB score: 4 (0–11) points). Apart from the classification criteria (MMSE score < 24 vs. ≥24), subsamples with and without CI differed with respect to frailty-, functional-, motor-, and psychological status (CFS, ADL, SPPB AES-C) indicating a reduced status in persons with CI but more apathetic symptoms in the group without CI (Table 1).

**Table 1:** Participant characteristics for the total study sample (*n* = 119), and subgroups according to cognitive status

Characteristics	(Mean (SD)/ n (%) / Median (range) Total group *n* = 119)	Non-CI (MMSE ≥24) *n* = 57	CI (MMSE< 24) *n* = 62	*P*-value*
Demographic factors				
Age, years	82.95 (6.20)	82.49 (6.54)	83.37 (5.89)	.488
Gender, female, n (%)	73 (63.5%)	38 (66.7%)	38 (61.3%)	.546
Health status				
BMI^a^	26.24 (13–48)	26.33 (16.98–48.04)	25.87 (13.77–37.42)	.480
Medications, number	10.22 (4.28)	10.07 (4.29)	10.40 (4.26)	.773
CFS, score^c^	6 (3–8)	6 (3–7)	6 (3–8)	**.012**
PPI, score	0 (0–5)	0 (0–5)	0 (0–4)	.501
No. of falls in the previous year^b^	1 (0–4)	1 (0–2)	1 (0–4)	.463
Cognitive status				
MMSE, score	23 (10–30)	27 (24–30)	19 (10–23)	**<.001**
Psychosocial status				
Short FES-I, score^b^	11(6–26)	11.5 (6–25)	10.5 (7–26)	.514
EQ-5D score	0.70 (0.96–1.00)	0.79 (0.06–1.00)	0.64 (0.06–1.00)	.237
AES-C, score^b^	25 (5–35)	26 (6–35)	22 (5–35)	**.017**
Motor-functional status				
ADL Barthel score^c^	75 (5–100)	80 (25–100)	65 (5–100)	**.009**
SPPB, score^a^	4 (0–11)	5 (0–11)	3 (0–10)	**.026**
Gait velocity (s)^d^	7.63 (4.75)	7.13 (4.74)	8.15 (4.76)	.300
Physical activity				
PA (mean number of steps per day)	704 (0–7095)	813 (1.5–6772)	690 (0–7095)	.293

Presented are patient characteristics for the study sample description for the total group and according to cognitive status. Total sample included 119 participants, however, single measures are lacking for some individuals, including ^a^ *n* = 110 participants, ^b^ *n* = 112 participants, ^c^ *n* = 113 participants, ^d^ *n* = 94 participants. *p-values are given for Mann-Whitney *U* tests and *t-*tests as appropriate

*Abbreviations*: *AES-C* Apathy Evaluation Scale – Clinical version, *BMI* Body Mass Index, *CFS* Clinical Frailty Scale, *MMSE* Mini-Mental State Examination, *PPI* Present Pain Intensity Scale, *SPPB* Short-Physical-Performance-Battery, *Short FES-I* Short Falls Efficacy Scale-International, 7-item version, *VRS* Verbal rating scale, *EQ-5D* European health-related quality of life questionnaire (EuroQol-questionnaire, EQ-5D-3L), *n* Numbers, *CI* Cognitive impairment, *SD* Standard deviation, *m/s* Meters per second, *PA* Physical activity

### Description of life-space mobility

The mean total score for the LSA-IS of 12.7 ± 9.0 (range: 0–48 points) indicated a largely restricted LSM for the total group. Restriction of life-space was also confirmed for all sub-scores such as the LSA-IS-M for maximal life-space with technical and personal support: (2.2 ± 1.0), the LSA-IS-E as achieved with supportive devices (1.7 ± 1.2), and the independent life-space (LSA-IS-I), achieved without human or technical assistance (0.4 ± 1.0). Results differed significantly between persons with and without CI for the total score and the LSA-IS-E, but not for the LSA-IS-M and the LSA-IS-I (Table 2).

**Table 2:** Baseline LSA status for the LSA-IS composite score and sub-scores

LSA-Variables	Total group *n* = 117^a^	Non-CI *n* = 57	CI *n* = 60	*P*-value **
	Mean (SD)	Mean (SD)	Mean (SD)	
LSA-IS-T total score	12.72 (9.00)	14.94 (10.34)	10.62 (6.96)	**.035**
LSA-IS-M maximal score	2.17 (.096)	2.35 (1.04)	2.00 (0.84)	.084
LSA-IS-E equipment-assisted score	1.74 (1.16)	2.05 (1.16)	1.45 (1.10)	.**008**
LSA-IS-I independent score	0.44 (1.99)	0.51 (1.14)	0.37 (0.86)	.546

Presented are results for the total group and sub-groups according to cognitive status

*Abbreviations*: *LSA-IS* Life-space assessment for institutionalized settings, *−T* Total score, *−M* Maximal life-space, *E* Equipment assisted life-space, *I* Independent life-space

**p-values are given for Mann Whitney U tests for differences between persons without cognitive impairment (Non-CI; *n* = 57), and persons with cognitive impairment (CI; *n* = 60) at baseline. ^a^*n* = 2 were excluded, as these persons were transferred for diagnostic reasons during the relevant time period

### Construct validity

In the total sample, the LSA-IS-T (total score) showed significant moderate associations to most variables documenting demographic and health status (age, BMI, number of medication, frailty status), cognitive status (MMSE), or psychosocial status (FES-I, EQ-5D, AES-C). Other variables such as gender, history of falls, and pain were not associated with LSA-IS (Table 3). Motor-functional status and activity behavior stood out with significant associations in all parameters especially so for variables related to activity behavior indicated by higher associations to LSM.

**Table 3:** Construct validity for the LSA-IS total score and sub-scores

	Total group				Non-CI				CI			
	LSA-IS-T total score	Subscores			LSA-IS-T total score	Subscores			LSA-IS-T total score	subscores		
		LSA-IS-M maximal	LSA-IS-E equipment-assisted	LSA-IS-I independent		LSA-IS-M maximal	LSA-IS-E equipment-assisted	LSA-IS-I independent		LSA-IS-M maximal	LSA-IS-E equipment-assisted	LSA-IS-I independent
Demographic factors												
Age	−.295^**^	−.291^**^	−.194^*^	−.195^*^	−.415^**^	−.434^**^	−.320^*^	−.282^*^	−.164	−.137	−.055	−.103
Gender^a^	.000	−.009	.105	−.027	−.009	.002	.045	−.022	.032	.016	.209	−.026
Health status												
BMI	−.210^*^	−.189	−.136	−.139	−.233	−.115	−.076	−.046	−.217	−.248	−.191	−.241
No. of medication	−.302^**^	−.302^**^	−.292^**^	−.239^**^	−.213	−.256	−.164	−.127	−.382^**^	−.344^**^	−.421^**^	−.350^**^
CFS	−.387^**^	−.281^**^	−.391^**^	−.139	−.350^*^	−.282^*^	−.394^**^	−0.184	−.369^**^	−.249	−.314^*^	−.042
No of falls in the previous year	−.144	−.111	−.120	−.120	−.023	−.116	.036	.015	−.244	−.087	−.259	−.263
PPI	−.055	−.056	−.034	.077	.023	.002	.064	.103	−.170	−.172	−.187	.022
Cognitive status												
MMSE	.213^*^	.202^*^	.267^**^	−.003	.262^*^	.315^*^	.177	.015	−.078	−.053	.066	−.221
Psychosocial status												
EQ-5D score	.403^**^	.268^**^	.408^**^	.182	.186	.135	.094	.307^*^	.154	.189	.144	−.113
AES-C score	.225^*^	.219^*^	.256^**^	−.069	.244	.199	.276^*^	−.192	.167	.219	.161	−.048
Short-FESI score	−.299^**^	−.208^*^	−.259^**^	−.113	−.277^*^	−.178	−.214	−.016	−.357^**^	−.250	−.363^**^	−.245
Motor-functional status												
ADL Barthel, score	.579**	.389**	.650**	.231*	.527^**^	.365^**^	.574^**^	.181	.588^**^	.374^**^	.683^**^	.207
SPPB, score	.575^**^	.330^**^	.591^**^	.454^**^	.590^**^	.374^**^	.566^**^	.433^**^	.513^**^	.248	.583^**^	.428^**^
Gait Velocity, s	−.518^**^	−.359^**^	−.481^**^	−.371^**^	−.493^**^	−.299^*^	−.435^*^	−.355^*^	−.495^**^	−.406**	−.459^*^	−.332^*^
Physical activity												
Duration of lying	−.381^**^	−.450^**^	−.372^**^	.043	−.513^**^	−.589^**^	−.560^**^	−.019	−.052	−.073	−.063	.152
Duration of activity	.538^**^	.399^**^	.600^**^	.327^**^	.448^**^	.307	.494^**^	.471^**^	.565^**^	.309	.575^**^	.416^*^
Duration of gait	.716^**^	.555^**^	.598^**^	.468^**^	.677^**^	.519^**^	.552^**^	.460^**^	.776^**^	.549^**^	.675^**^	.586^**^
Number of steps	.713^**^	.567^**^	.588^**^	.457^**^	.665^**^	.522^**^	.543^**^	.439^**^	.756^**^	.553^**^	.650^**^	.575^**^

Presented are Spearman rank correlation coefficients (r_s_), except for gender ^a^ point-biserial correlation coefficients (r_pb_)

*Abbreviations*: *ADL* Activities of daily living, *AES-C* Apathy Evaluation Scale – Clinical version, *BMI* Body mass Index, *CFS* Clinical Frailty Scale, *EQ-5D score* EuroQol-questionnaire score EQ-5D-3L version, *LSA-IS* Life-Space Assessment for institutionalized settings, *LSA-IS-C* Composite life-space mobility, *LSA-IS-M* Maximum life-space, *LSA-IS-E* Maximum life-space with equipment, *LSA-IS-I* Maximum independent life-space, *MMSE* Mini-Mental State Examination, *PPI* Present Pain Intensity Scale, *SPPB* Short-Physical Performance Battery, *Short FES-I* Short Falls-Efficacy-Scale. Correlations coefficients (r): small (*r* = 0.1–0.3), moderate (*r* = 0.3–0.5), or high (*r* > 0.5). *p < .05. **p < .01; b point-biserial correlation coefficients (rpb)

On average a trend for lower associations is visible for the sub-score LSA-IS-I (independent score) as compared to the total score and other sub-scores throughout all samples.

When classified into persons with intact and impaired cognition, results of the total group were confirmed for most associations, while descriptive variables and variables related to health status were less associated. Some variables differed as age, cognitive status, and duration of lying were only significantly associated to LSM in the group of cognitively intact persons, while the number of medications was only significantly correlated in the group of persons with CI (Table 3).

### Test–retest reliability

ICCs between the two LSA-IS assessments indicated fair to excellent test–retest reliability for all LSA-IS scores, with ICCs ranging from .412–.799 except for 1 result (LSA-IS-M, .285, subgroup with CI). Results for test-retest reliability were comparable in both groups according to cognitive status. Within LSA-IS scores, the LSA-IS-M (maximal) score presents with an on average lower reliability as compared to all other scores while the LSA-IS-I (independent) stood out with excellent results in all samples (Table 4).

**Table 4:** Test – retest reliability for total group and subgroups

Variable	First test	Retest	ICC	95% CI
	Mean (SD)			
Total group (*n* = 76)				
LSA-IS-T	18.42 (10.21)	19.20 (10.91)	.704	.570–.801
Subscores				
LSA-IS-M	2.74 (0.93)	2.79 (0.94)	.412	.208–.582
LSA-IS-E	2.32 (1.16)	2.34 (1.23)	.741	.620–.827
LSA-IS-I	0.64 (1.10)	0.80 (1.34)	.799	.701–.868
Non-CI/Subsample with intact cognition (*n* = 36)				
LSA-IS-T	19.29 (11.29)	21.72 (12.50)	.715	.511–.843
Subscores				
LSA-IS-M	2.81 (0.92)	2.86 (0.96)	.558	.288–.747
LSA-IS-E	2.56 (1.06)	2.67 (1.04)	.671	.445–.817
LSA-IS-I	0.69 (1.17)	0.83 (1.34)	.798	.641–.891
CI/Subsample with impaired cognition (*n* = 40)				
LSA-IS-T	17.64 (9.19)	16.93 (8.80)	.676	.467–.814
Subscores				
LSA-IS-M	2.68 (0.94)	2.73 (0.93)	.285	−.023–.544
LSA-IS-E	2.10 (1.22)	2.05 (1.32)	.766	.602–.869
LSA-IS-I	0.60 (1.06)	0.78 (1.35)	.805	.662–.892

Presented are results of test-re test reliability for the total group and sub-scores according to cognitive status

*Abbreviations*: *CI* Confidence interval, *LSA-IS* Life-Space Assessment for institutionalized settings, *LSA-IS-T* Total life-space mobility, *LSA-IS-M* Maximum life-space, *LSA-IS-E* Maximum life-space with equipment, *LSA-IS-I* Maximum independent life-space; ICCs were interpreted as poor (< 0.4), fair to good (≥ 0.4 ≤ 0.75), and excellent (> 0.75)

### Sensitivity to change

All LSA-IS scores differed over the treatment period (*p* ≤ .001), indicating a global sensitivity to change of the assessment method. Improvements of LSM as documented by SRMs during the hospital stay were found across all LSA-IS scores for the total group, with the highest SRM for the LSA-IS-T (.806), while LSA-IS-I sub-scores reached lower SRMs (.326–.667). The two groups according to cognitive status achieved comparable results with the highest value for the total score (cognitively intact persons: .812, persons with CI: .833), and values for sub-scores ranging from .383–.812 for persons with intact cognition and .269–.833 for persons with CI. Across all samples, the LSA-IS-I (independent score) showed the least responsiveness during hospital stay, while results of the total score achieved SRMs >.800 in all samples indicating large responsiveness (Table 5).

**Table 5:** Sensitivity to change of the LSA-IS total score and sub-scores

Variables	Test at admission	Test at discharge	*P*-value*	SRM	Adjusted SRM
	Mean (SD)				
Total sample (*n* = 69)					
LSA-IS-T	12.43 (8.82)	19.17 (11.12)	<.001	0.696	0.806
Subscores					
LSA-IS-M	2.22 (1.00)	2.80 (1.01)	<.001	0.545	0.667
LSA-IS-E	1.68 (1.14)	2.29 (1.21)	<.001	0.574	0.647
LSA-IS-I	0.46 (1.11)	0.78 (1.33)	<.001	0.292	0.326
Subsample with intact cognition (*n* = 33)					
LSA-IS-T	14.20 (9.62)	21.89 (12.77)	<.001	0.700	0.812
Subscores					
LSA-IS-M	2.30 (0.98)	2.91 (1.01)	<.001	0.572	0.701
LSA-IS-E	1.97 (1.08)	2.67 (0.99)	<.001	0.648	0.786
LSA-IS-I	0.55 (1.23)	0.94 (1.41)	<.001	0.353	0.383
Subsample with cognitive impairment (*n* = 36)					
LSA-IS-T	10.82 (7.81)	16.67 (8.82)	<.001	0.701	0.833
Subscores					
LSA-IS-M	2.14 (1.02)	2.69 (1.01)	<.001	0.514	0.631
LSA-IS-E	1.42 (1.16)	1.94 (1.31)	<.001	0.500	0.548
LSA-IS-I	0.39 (0.96)	0.64 (1.25)	.004	0.232	0.269

Presented are results for sensitivity to change for effects of early rehabilitation during hospital stay

*Abbreviations*: *SD* Standard deviation, *SRM* Standardized response mean, *LSA-IS* Life-Space Assessment for institutionalized settings, *LSA-IS-T* Total life-space mobility, *LSA-IS-M* Maximum life-space, *LSA-IS-E* Maximum life-space with equipment, *LSA-IS-I* Maximum independent life-space; Adjusted SRMs are classified as < 0.2 = trivial, ≥0.2 < 0.5 = small, ≥0.5 < 0.8 = moderate, ≥0.8 = large

*difference between admission and discharge

### Feasibility

No participant objected to the assessment procedure and data documentation was comprehensive, with no missing responses for any LSA-IS item (100% completion rate). Mean completion time to assess the LSA-IS was brief with 3.2 ± 1.2 min (range 1–11) and not significantly different between subgroups by cognitive status (CI: 3.0 ± 0.9 vs. non-CI: 3.4 ± 1.5). For the total score no relevant ceiling (no participant) or floor effects (*n* = 3 (2.6%) occurred [35]. None of the sub-scores presented ceiling effects, however, the sub-scores LSA-IS-E and the LSA-IS-I showed relevant floor effects (LSA-IS-E: 19.3%; LSA-IS-I: 79.0%) in this population of vulnerable patients during hospital stay.

## Discussion

The LSA-IS (total score) demonstrated good test–retest reliability, high sensitivity to change, while construct validity was appropriate depending on relative construct association as hypothesized. Results of LSA-IS sub-scores confirmed most results of the total score, while results of persons with and without CI did not differ for most analyzed variables. A very high completion rate and a brief completion time indicated excellent feasibility for use in research or clinical routines.

### Construct validity

For construct validation we used construct variables out of different domains to allow a validation within a comprehensive Life Space Mobility model. We assumed different levels of associations for different domains based on their content association to LSM based on results of previous comparable studies [14, 16, 32] and in line with the comprehensive and well-established mobility model by Webber [6]. Present results of the construct validation fitted well with these a priori assumptions, indicating good construct validity of the assessment. The LSA-IS total score showed significant, moderate associations to most variables documenting demographic-, health-, cognitive-, and psychosocial status representing more “distant” domains to the LSA representing a behavioral activity measure As hypothesized, motor-functional status and activity behavior stood out with significant, higher associations for all construct parameters with all of them representing a common motor domain.

Two out of three sub-scores (LSA-IS-M, LSA-IS-E) followed the results for the total score, while a trend for lower associations was visible for the sub-score LSA-IS-I (independent score). We assume the weaker association to be caused by the lower incidence of independent, non-supported mobility in this multi-morbid, vulnerable sample during acute, ward-based medical care with a higher number of participants with low levels of LSA-IS-I.

Results of the construct validation also indicate that achieved good results were not different between subgroups according to cognitive status for most variables, indicating good validity also for persons with moderate to more advanced stages of CI, representing the majority of patients in geriatric hospitals as well as other institutionalized settings.

### Test-retest reliability

Test–retest reliability was good to excellent in the total score as well as two out of three sub-scores, indicating highly stable results for the LSA-IS in general. Results are in line with a trend for lower reliability as compared to previous validation studies for community dwelling older persons documented by ICCs [14, 16]. The lower test-re-test reliability for the sub-score maximal life-space is in line with a trend for lower reliability as compared to a previous validation study for community dwelling older persons documented by ICCs [16] and may be caused by the fact that maximal life-space, including outer ranges of mobility such as cafeteria visits or even outdoor visits beyond into the neighboring surrounding (e.g. hospital garden), are more infrequent and random in vulnerable, institutionalized persons. Occurrence of such events heavily depend on external support and are therefore less reliable when tested in a short period of time, but do not necessarily indicate a lower biometrical quality of the evaluation method. However, the low reliability of this sub-score may indicate a limitation for this specific hospital setting.

### Sensitivity to change

All LSA-IS scores significantly increased during the relatively short assessment period within an intervention (early ward-based rehab) which was not specifically tailored to achieve LSM changes, indicating a global sensitivity to change of the assessment method. Specific modification of the LSA-IS, such as framing of the observation period, supported the good responsibility. Apart from supporting recall in persons with memory deficits, relevant for all institutional settings, the short observation period as used for the LSA-IS, represented a precondition to document sensitivity to change in hospital or rehab settings within the given limited time frame of therapeutic interventions.

Other life-space assessment validation did predominantly not include this biometrical measure which is highly relevant to document efficacy of interventions in research or clinical routines. The two comparable studies used different statistical strategies. While Baker used a dichotomized descriptive analysis without statistical analysis and a longitudinal, observational design demonstrating adequate responsiveness [16], Ullrich reported comparable results to the present study for effects of an RCT on activity promotion to document good sensitivity to change [14].

In the present study sensitivity to change as documented by SRMs was large for the main total score summarizing all changes for all independence levels and areas of mobility. Moderate effects for the sub-scores LSA-IS-M and LSA-IS-E documented effects of routine hospital-based rehab, leading to relevant changes to extent mobility by using technical or personal support. The sub-score with the least responsiveness (LSA-IS-I) was relevant for only a minority of high functioning persons not using such support, which is almost mandatory for ambulation of multi-morbid persons within hospitals [40]. The somewhat lesser responsiveness may therefore rather be an indicator of the very low functional status of the study sample than a methodological limitation of the evaluation method. As with other biometrical domains in this study, no relevant differences were documented between subgroups according to cognitive status, indicating good responsiveness of the LSA-IS also in persons with CI. Results, as achieved in this study may mirror the special focus of the development of the LSA-IS on persons with CI, that require a specific approach due to their special needs [13, 14, 41].

### Feasibility

The 100% completion rate with no missing responses documented an excellent feasibility of the LSA-IS even in multi-morbid, vulnerable persons during acute medical, ward-based treatment. The specific component of the questionnaire such as the interview-based interrogation, the interview technique tailored to the study sample including persons with moderate to more advanced stages of CI, as in previous successful validation studies [13, 14] and the highly organized setting with restricted degrees of freedom for mobility following standardized routines, which are easy to recall and structure by interview, may have helped to achieve this extraordinary result. The LSA-IS completion time of 3.2 min is very brief allowing its use in research as well as clinical routines with only very little resources and no risk of overtaxation of interviewed persons.

As in comparable questionnaires [20] or sensor-based LSA [10], the LSA-IS includes categories extending beyond the institutions covering the clinically relevant transition from protected indoor to demanding outdoor activity.

In the present study floor effects occurred in two sub-scores with focus on equipment-assisted or independent life-space. In a clinical acute setting with multi-morbid, vulnerable persons, most of those not able to independently move without technical or personal support and with institutional activity restrictions to stay indoors, such a result is to be expected. On the other hand, as a logical consequence, the main total score and the sub-score including technical or personal support did not have such floor effects and none of the scores showed ceiling effects as documented in other LSM assessment validation studies which reported on this issue [14, 16]. It is noteworthy that good feasibility could be achieved also in the group with moderate to more advanced stages of CI, with specific limitations for a questionnaire-based assessment.

### Limitations

Although the LSA-IS has been developed for generic use in comparable institutional settings, formally the present validation is focused on ward-based acute geriatric care and generalizability of its’ psychometric properties will have to be additionally confirmed for other settings.

## Conclusions

The results of this study demonstrate good validity, reliability, sensitivity to change, and feasibility of the newly developed LSA-IS in geriatric patients without and with moderate to advanced stages of CI during acute, in-hospital medical treatment. Based on the high comparability of organizational structures and populations, we consider the use of the instrument feasible in other comparable institutionalized settings such as other in-house medical care, rehabilitation centers, or in nursing homes settings.

## Supplementary Information

**Additional file 1.** Life-Space Assessment in Institutionalized Settings (LSA-IS). (documentation form)

**Additional file 2.** Life-Space Assessment for Persons in institutionalized settings (LSA-IS) – User Manual.

**Additional file 3.** COSMIN Study Design checklist for Patient-reported outcome measurement instruments.

## Data Availability

Availability was only given to participants (for their own information) and specific members of the study. The datasets generated and/or analysed during the current study are not publicly available as the ethical vote did not include open data access, but are available from the corresponding author on reasonable request.

## Abbreviations

AES-C: Apathy Evaluation Scale – Clinical version
BMI: Body Mass Index
CFS: Clinical Frailty Scale
CI: Cognitive impairment
EQ-5D: European health-related quality of life questionnaire (EQ-5D-3L)
LSA-CI: Life-Space Assessment in Persons with cognitive impairment
LSA-IS: Life-Space Mobility in Institutionalized Settings
LSM: Life-space mobility
n: Numbers
NHLSD: Nursing Home Life-Space Diameter
MMSE: Mini-Mental State Examination
PA: Physical activity
PPI: Present Pain Intensity Scale
SD: Standard deviation
SPPB: Short-Physical-Performance-Battery
Short FES-I: Short Falls Efficacy Scale-International, 7-item version
UAB-LSA: University of Alabama at Birmingham – Life-Space Assessment
VRS: Verbal rating scale
