# Supplementary material for: Validation of the interview-based life-space assessment in institutionalized settings (LSA-IS) for older persons with and without cognitive impairment
Source: BMC Geriatr. 2020 Dec 10;20:534. doi: 10.1186/s12877-020-01927-8 (PMC7726908; doi:10.1186/s12877-020-01927-8)
Supplement: Supplementary file 1 — Additional file 1. Life-Space Assessment in Institutionalized Settings (LSA-IS). (documentation form) [file 12877_2020_1927_MOESM1_ESM.docx]

Additional File 1:

**Life-Space Assessment in Institutionalized Settings (LSA-IS)**

| Name: | | | Date: | | |
| --- | --- | --- | --- | --- | --- |
| **All questions refer to your activities within the previous day.** | | | | | |
| **Life-space level** | | **Frequency** | | **Independence** | **Score** |
| **Have you been yesterday active…** | | **How often did you get there?** | | **Did you use aids or equipment? Did you need help from another person?** | Level x Frequency x Independence |
| *Level 1…*  **within your room?** | 1 = Yes  0 = No | 1 = 1 × /day  2 = 2-3 × /day  3 = 4-5 × /day  4 = >5 × /day | | 1 = Personal assistance  1.5 = Equipment only  2 = No equipment or personal assistance | _________________  *Level 1 scores* |
| ***Score*** | _____ × | _____ × | | _____ = |  |
| *Level 2…*  **...out of your room but within the ward?** | 2 = Yes  0 = N0 | 1 = 1 × /day  2 = 2-3 × /day  3 = 4-5 × /day  4 = >5 × /day | | 1 = Personal assistance  1.5 = Equipment only  2 = No equipment or personal assistance | _________________  *Level 2 scores* |
| ***Score*** | _____ × | _____ × | | _____ = |  |
| *Level 3…*  **Out of the ward but within the institution building (other wards, cafeteria, chapel)?** | 3 = Yes  0 = No | 1 = 1 × /day  2 = 2-3 × /day  3 = 4-5 × /day  4 = >5 × /day | | 1 = Personal assistance  1.5 = Equipment only  2 = No equipment or personal assistance | ________________  *Level 3 scores* |
| ***Score*** | _____ × | _____ × | | _____ = |  |
| *Level 4…*  **Out of the institution building, but within the outdoor area of the institution (garden, park)?** | 4 = Yes  0 = No | 1 = 1 × /day  2 = 2-3 × /day  3 = 4-5 × /day  4 = >5 × /day | | 1 = Personal assistance  1.5 = Equipment only  2 = No equipment or personal assistance | _________________  *Level 4 scores* |
| ***Score*** | _____ × | _____ × | | _____ = |  |
| *Level 5…*  **out of the outdoor area of your institution?** | 5 =Yes  0 = No | 1 = 1 × /day  2 = 2-3 × /day  3 = 4-5 × /day  4 = >5 × /day | | 1 = Personal assistance  1.5 = Equipment only  2 = No equipment or personal assistance | _________________  *Level 5 scores* |
| ***Score*** | _____ × | _____ × | | _____ = |  |
| **Total score**  **(LSA-IS-T)** | | | | | _________________  *Sum* |
| Max. Level  (LSA-IS-M) | | | | | ___________ |
| Max. equipment-assisted Level without personal assistance  (LSA-IS-E) | | | | | ___________ |
| Max. independent Level without equipment and without personal support  (LSA-IS-I) | | | | | ___________ |
