# Supplementary material for: Validation of the interview-based life-space assessment in institutionalized settings (LSA-IS) for older persons with and without cognitive impairment
Source: BMC Geriatr. 2020 Dec 10;20:534. doi: 10.1186/s12877-020-01927-8 (PMC7726908; doi:10.1186/s12877-020-01927-8)
Supplement: Supplementary file 2 — Additional file 2. Life-Space Assessment for Persons in institutionalized settings (LSA-IS) – User Manual. [file 12877_2020_1927_MOESM2_ESM.docx]

Additional File 2:

**Life-Space Assessment for Persons in**

**institutionalized settings (LSA-IS)**

**–**

**User Manual**

**Introduction**

The Life-Space Assessment for institutionalized settings (LSA-IS) has been developed on the basis of the Nursing Home Life-Space Diameter ([20](file:///D:\Programs\ProductionJournal\Temp\ADDITIONAL%20FILES%20Manuscript%20Validation%20LSA-IS%20R2R3.docx#_ENREF_20)), the University of Alabama at Birmingham – Life Space Assessment ([16](file:///D:\Programs\ProductionJournal\Temp\ADDITIONAL%20FILES%20Manuscript%20Validation%20LSA-IS%20R2R3.docx#_ENREF_16)) and the Life-Space Assessment for Persons with Cognitive Impairment ([14](file:///D:\Programs\ProductionJournal\Temp\ADDITIONAL%20FILES%20Manuscript%20Validation%20LSA-IS%20R2R3.docx#_ENREF_14)) to assess life-space mobility in older persons in institutionalized settings. It assesses mobility within geographically defined areas (room, ward, whole indoor facility, outdoor area of the facility, and areas beyond the area of the facility e.g. public area/neighborhood, town and beyond), including the frequency of movement and the need of support by equipment or other persons. The assessment is supported by a specifically developed interview-technique to gather correct and comprehensive information despite potential negative influence of cognitive impairment, acute medical problems, or recent hospitalization.

**Target population**

The target population includes older persons in institutions, such as hospitals, rehabilitation clinics, nursing homes or other care institutions. These persons might suffer from multi-morbidity, acute medical syndromes, cognitive impairment, or disorientation due to recent institutionalization/hospitalization.

**Preparation for administration**

No specific extended training is needed for health professionals to administrate the LSA-IS and the assessment does not require any additional material for administration other than the questionnaire and a pencil.

**Administration guidelines**

The LSA-IS represents an interview-based method directly addressing the target sample of older, multi-morbid, physically impaired and/or cognitively impaired persons. It needs to be conducted with the subject alone excluding potential distraction. In case a roommate, spouse or carer is present, he/she should not take part in the assessment procedure and should not respond to questions.

The assessment should be conducted in a quiet, undisturbed environment. It is preferable to administer the LSA-IS in the subject`s current residence/room, thus the interviewer is able to refer to the actual environment while asking for each life-space level and equipment/assistive device used. Knowledge about the living situation is also useful to check the accuracy of the participant’s answers and thus to ask appropriate queries. The interviewer should actively ask for obvious motor impairments (e.g., while standing up or walking) and for the equipment used (e.g., cane or walker is to be shown) to achieve a realistic impression of the mobility status**.**

The interview has to be conducted face-to-face and interview-based to exclude insufficient completion of the assessment and overtaxing of the persons’ abilities to cope and respond. This conversational approach includes comprehensive verbal information and feedback during the assessment to eliminate miscomprehension or ambiguities and prevents fear of failure in comprehension and recall of the person ([13](file:///D:\Programs\ProductionJournal\Temp\ADDITIONAL%20FILES%20Manuscript%20Validation%20LSA-IS%20R2R3.docx#_ENREF_13), [14](file:///D:\Programs\ProductionJournal\Temp\ADDITIONAL%20FILES%20Manuscript%20Validation%20LSA-IS%20R2R3.docx#_ENREF_14)).

**Step 1:** The interview should start with a short explanation of the objective and the approximate length of the assessment.

**Step 2:** It is important to clearly define the beginning of the measurement period and the end of the measurement period (the previous day). Start every question with defining the observation period: “Yesterday, …”. To prevent confusion and unclear statements by the participant, it is appropriate to assess each day segment structured by typical daily routines such as meals, standardized therapy or care.

**Step 3:** Ask for each specific life-space level, starting with life-space level 1 and progressively continuing with the next life-space levels without skipping one level.

Habitual activities of a persons should be included and used as “anchors” to segment the day and support the recall (waking up, going to bed, having meals are main anchors usually taking place at fixed time points in institutionalized settings, which can be amended by others/ individual routines such as going to the bathroom, getting newspaper, watching TV, or regular social activities such as sport group or therapy, etc.).

Additionally, the interviewer should ask for typical activities during the observation period, if they are not mentioned by the person (therapy, care, visits by family or friends, visiting cafeteria or bistro, etc.) to improve the completeness of the report. Closed questions may be appropriate to avoid cognitive overload.

Keep in mind that the LSA-CI is a measure of actual mobility - what the subject has done and not what the subject might be doing - within the previous day. For accuracy check of the scoring, it is not possible to reach a higher life-space level without reaching the previous life-space level, this should be clarified in case of any contradictory information.

**Step 4:** The end of each life-space level assessment includes a rehearsel of frequency and assistance specific for the respective level by the interviewer. The administrator should take time to rehearse the results once again with the participant to ensure that statements are understood correctly.

**Examples for questioning and rehearsel:**

Level 1: *Yesterday, did you move around within this room/ the room where you sleep? Have you been ill or spend the day in bed? Did you use equipment to leave your bed? While going to the table or to the wardrobe (etc.), did you use a cane/rollator…?*

Rehearsel Level 1: *Ok, yesterday, you moved around in your room more than five times without assistance. Is this correct?*

Level 2: *Yesterday, have you left your room and have you been somewhere within this ward such as the common room, therapy room, doctor´s room … (*refer to the specific surrounding of the subject`s ward)*? How often have you been there? Did you use a rollator/cane etc.? Was there another person that helped you doing that?*

Rehearsel Level 2: *Let me summarize: Yesterday, you left your room three times to go to the common room for meals while using your cane. Is this correct? Any other events you spent outside your room that come to your mind now that you think about it again?*

Level 3: *During the past day, have you been to other places in this hospital outside this ward? For example in the cafeteria, bistro, chapel or other* (choose appropriate wording and location)*? For example walking around in the hospital halls or visiting other residents/patients* (if appropriate)*? How often did you leave the ward? I have seen the cafeteria/bistro/chapel etc., did you go there? Do you need help for this by a nurse/ carer etc. or do you use your cane/rollator etc.?*

Rehearsel Level 3: *Yesterday, you left the ward once. You had a visit by your daughter in the afternoon and you went to the cafeteria. Is this correct?*

Level 4: *Yesterday, have you been to places outside the facility? Have you visited the garden/park? How often did you get there? Do you walk there? Do you use a cane/rollator? Do you need help by another person for physical assistance or for safety reasons?*

Rehearsel Level 4: *Let me repeat, you left the hospital once to walk around in the hospital park. You used your rollator to walk, but felt unsafe without assistance by your son, who supported you taking your arm. Is this correct?*

Level 5: *Yesterday, have you left* *the outdoor area/garden/park of the facility/hospital/nursing home and have been walking/driving around in the neighborhood/town etc.? How often have you been there? How did you get there* (use transportation mode that was given before: walking independent or with equipment, with assistance by another person)*?*

Rehearsel Level 5: *You did not leave the facility´s area. Is this correct?*

**Invalid assessments and exclusion of assessments:**

In case of obvious unrealistic reports and implausible statements by the person, data has to be excluded.

Examples for exclusion of assessment:

- Participant reports mobility without assistance, but is obviously unable to walk without assistive device
- Participant states not to have left the bed but is actually sitting in another room
- Carer or other person disagrees with statements afterwards

For Information Please Contact:

Prof. Dr. Klaus Hauer

Department of Geriatrics, University of Heidelberg

phone: +49 6221 / 319-1783

email: khauer@bethanien-heidelberg.de

Agaplesion Bethanien Hospital,

Rohrbacher Str. 149,

69126 Heidelberg, Germany

References:

Baker, P. S., Bodner, E. V., & Allman, R. M. (2003). Measuring life-space mobility in community-dwelling older adults. J Am Geriatr Soc, 51(11), 1610-1614. doi: 10.1046/j.1532-5415.2003.51512.x

Hauer, K., Lord, S. R., Lindemann, U., Lamb, S. E., Aminian, K., & Schwenk, M. (2011). Assessment of physical activity in older people with and without cognitive impairment. J Aging Phys Act, 19(4), 347-372. doi: 10.1123/japa.19.4.347

Tinetti, M. E., & Ginter, S. F. (1990). The nursing home life-space diameter. A measure of extent and frequency of mobility among nursing home residents. J Am Geriatr Soc, 38(12), 1311-1315.

Ullrich, P., Werner, C., Bongartz, M., Kiss, R., Bauer, J., & Hauer, K. (2019). Validation of a Modified Life-Space Assessment in Multimorbid Older Persons With Cognitive Impairment. Gerontologist, 59(2), e66-e75. doi: 10.1093/geront/gnx214
