# Supplementary material for: Validation of the interview-based life-space assessment in institutionalized settings (LSA-IS) for older persons with and without cognitive impairment
Source: BMC Geriatr. 2020 Dec 10;20:534. doi: 10.1186/s12877-020-01927-8 (PMC7726908; doi:10.1186/s12877-020-01927-8)
Supplement: Supplementary file 3 — Additional file 3. COSMIN Study Design checklist for Patient-reported outcome measurement instruments. [file 12877_2020_1927_MOESM3_ESM.docx]

Additional File 3

**COSMIN Study Design checklist for Patient-reported outcome measurement instruments**

*P Mokkink, DL Patrick, J Alonso, LM Bouter, H CW de Vet, & CB Terwee*

- Version July 2019 -

General recommendation for the design of a study on measurement properties

The box General recommendations for designing a study on measurement properties is relevant for all studies on measurement properties. The aim of a study evaluating a measurement property of a PROM is to investigate (one or more aspects of) the quality of the PROM at issue. These studies require a clear research aim (i.e. referring to the measurement properties of interest), a clear description of the PROM and a clear description of the study population. The quality of a PROM should be determined in the target population in which the PROM will be used, because the results of studies on measurement properties depend on the sample included in the study.

| **General recommendations for the design of a study on measurement properties** |  |  |  |  |  |
| --- | --- | --- | --- | --- | --- |
|  | **very good** | **adequate** | **doubtful** | **inadequate** | ***Reported***  ***on page #*** |
| ***Research aim***   1. Provide a clear research aim, including (1) the name and version of the PROM, (2) the target population, and (3) the measurement properties of interest | Research aim clearly described |  |  | Research aim not clearly described | ***6 (line 8-12)*** |
| ***PROM*** |  |  |  |  |  |
| 1. Provide a clear description of the construct to be measured | Construct clearly described |  |  | Construct not clearly described | ***4 (line 23-26)*** |
| 1. Provide a clear description of the development process of the PROM, including a description of the target population for which the PROM was developed | Development  process clearly described |  | Development  process clearly described |  | ***8 (line 1-26)*** |
| 1. The origin of the construct should be clear: provide a theory, conceptual framework (i.e. reflective or formative model) or disease model used or clear rationale to define the construct to be measured | Origin of the construct clear |  | Origin of the construct not clear |  | ***-*** |
| 1. Provide a clear description of the structure of the PROM (i.e. the number of items and subscales included in the PROM, instructions given and response options) | Structure and scoring algorithm  clearly described |  |  | Structure and scoring algorithm not clearly described | ***8 (line 1-26), 9 (line 1-18)***  ***User Manual*** |
| 1. Provide a clear description of existing evidence on the quality of the PROM | Existing evidence on the quality of the PROM clearly described |  | Existing evidence on the quality of the PROM not clearly described |  | ***NA*** |
| 1. Provide a clear description of the context of use* | Context of use clearly described |  | Context of use not clearly described |  | ***8 (line 1-17)*** |
| ***Target population*** |  |  |  |  |  |
| 1. Provide a clear description of in- and exclusion criteria to select patients, e.g. in terms of disease condition and characteristics like age, gender, language or country, and setting (e.g. general population, primary care or hospital/rehabilitation care) | In- and exclusion criteria for patients clearly described |  |  | In- and exclusion criteria for patients not clearly described | ***6 (line 21-26)***  ***7 (line 1-4)*** |
| 1. Provide a clear description of the method used to select the patients for the study (e.g. convenience, consecutive, or random) | Method for patient selection clearly described |  | Method of patient selection not clearly described |  | ***6 (line 22-24)*** |
| 1. Describe whether the selected sample is representing the target population in which the PROM will be used in terms of age, gender, important disease characteristics (e.g. severity, status, duration) | Study sample representing the target population clearly described | Assumable that the study sample is representing the target population, but not clearly described | Unclear whether the study sample is representing the target population | Study will not be performed in a sample representing  the target  population | ***User Manual*** |

* The context of use refers to the intended application of the PROM (e.g. for research or clinical practice), to a specific setting for which the PROM was developed (e.g. for use in a hospital or at home) or to a specific administration mode (e.g. paper or computer‐administered). If the PROM was developed for use across multiple contexts, this should be described.

Abbreviations NA, not applicable; PROM, patient-reported outcome measure.

Measurement error and reliability

Measurement error and reliability can be calculated based on the same study design and data collection. Basically, two measurements are needed in a group of people who are all assumed to be stable on the construct to be measured. As the design and the data collected can be used for both measurement properties, we present the standards in one box. Only the statistical parameters are different. We strongly encourage researchers who use such a design to report measurement error in addition to a reliability parameter.

| **Measurement error and reliability** |  |  |  |  |  |  |
| --- | --- | --- | --- | --- | --- | --- |
|  | **very good** | **adequate** | **doubtful** | **inadequate** | **NA** | ***Reported***  ***on page #*** |
| ***Design requirements***   1. Use at least two measurements | At least two measurements |  |  | Only one measurement |  | ***10 (line 9-10)*** |
| 1. Enure that the administrations will be independent | Independent measurements | Assumable that the measurements will be independent | Doubtful whether the measurements will be independent | measurements NOT independent |  | ***10 (line 9-10)*** |
| 1. Ensure that the patients will be stable in the interim period on the construct to be measured | Patients will be stable (evidence provided) | Assumable that patients will be stable | Unclear if patients will be stable | Patients will NOT be stable |  | ***10 (line 9-10)*** |
| 1. Use an appropriate time interval between the two measurements, which is long enough to prevent recall, and short enough to ensure that patients remain stable | Time interval appropriate |  | Doubtful whether time interval is appropriate or time interval is not stated | Time interval NOT appropriate |  | ***10 (line 9-10)*** |
| 1. Ensure that the test conditions will be similar for the measurements (e.g. type of administration, environment, instructions) | conditions similar (evidence provided) | Assumable that test conditions similar | Unclear if test conditions will be similar | Test conditions will NOT be similar |  | ***10 (line 9-10)*** |
| 1. Perform the analysis in a sample with an appropriate number of patients (taking into account expected number of missing values) | ≥100 patients | 50-99 patients | 30-49 patients | <30 patients |  | ***Table 4*** |
| ***Statistical methods for measurement error*** |  |  |  |  |  |  |
| 1. For continuous scores: calculate the Standard Error of Measurement (SEM), Smallest Detectable Change (SDC) or Limits of Agreement (LoA) | SEM, SDC, or LoA will be calculated, and model or formula is clearly described* | SEM or SDC will be calculated, but model or formula of the SEM or SDC is not described or not optimal** |  | SEM will be calculated based on Cronbach’s alpha, or on SD from another population | Not applicable | ***-*** |
| 1. For dichotomous/nominal/ordinal scores: calculate the percentage (positive and negative) agreement | % positive and negative agreement will be calculated | % agreement will be calculated |  | % agreement will not be calculated | Not applicable | ***-*** |
| 1. Provide a clear description of how missing items will be handled | The way missing items will be handled is clearly described |  | The way missing items will be handled is not clearly described |  | Not applicable | ***-*** |
| ***Statistical methods for reliability*** |  |  |  |  |  |  |
| 1. For continuous scores: calculate an intraclass correlation coefficient (ICC) | will be calculated, and model or formula of the ICC is clearly described* | ICC will be calculated, but model or formula of the ICC not described or not optimal** | Pearson or Spearman correlation coefficient will be calculated | No ICC or Pearson or Spearman correlations calculated | Not applicable | ***11 (line 8-10)***  ***Table 4*** |
| 1. For dichotomous/nominal/ordinal scores: calculate kappa | Kappa will be calculated |  |  | No kappa will be calculated | Not applicable | ***-*** |
| 1. For ordinal scores: calculate a weighted kappa | Weighted Kappa calculated and weighting scheme is described |  | Unweighted Kappa will be calculated or not described if Kappa will be weighted |  | Not applicable | ***-*** |
| 1. Provide a clear description of how missing items will be handled | The way missing items will be handled is clearly described |  | The way missing items will be handled is not clearly described |  |  | ***-*** |

* The model (i.e. one-way random effect model or two-way random or mixed effect model), type (i.e. for single or multiple measurement) and definition (i.e. for consistency or absolute agreement) of the ICC that will be calculated is appropriately chosen and described (see 11); ** ICC formula does not correspond to the research question

**Hypotheses testing for construct validity**

As no ‘gold standards’ exist for PROMs, the commonly used way to investigate validity of PROMs is to test hypotheses about 1) expected relationships with other outcomes measures of good quality (Part A), and/or 2) expected differences between relevant groups (Part B). It is of major importance to define hypotheses in advance when assessing construct validity of a PROM, to enable the authors to draw unbiased conclusions after data collection and analyses.

| **Hypotheses testing for construct validity**  **A. Comparison with other outcome measurement instruments (convergent validity)** |  |  |  |  |  |  |  |
| --- | --- | --- | --- | --- | --- | --- | --- |
|  | **very good** | | **adequate** | **doubtful** | **inadequate** | **NA** | ***Reported***  ***on page #*** |
| ***Design requirements***   1. Formulate hypotheses about expected relationships between the PROM under study and other outcome measurement instrument(s) | Hypotheses formulated including the expected direction and magnitude of the correlations stated | |  | Hypotheses vague or not formulated but possible to deduce what was expected | Unclear what is expected |  | ***11 (line 4-7)*** |
| 1. Provide a clear description of the construct(s) measured by the comparator instrument(s) | Construct(s) measured by the comparator instrument(s) is/are clearly described | |  |  | Construct(s) measured by the comparator instrument(s) is/are not clearly described |  | ***7 (line 6-18)***  ***10 (line 1-7)*** |
| 1. Use comparator instrument(s) with sufficient measurement properties | Sufficient measurement properties of the comparator instrument(s) in a population similar to the study population | | Sufficient measurement properties of the comparator instrument(s) but not sure if these apply to the study population | Some information on measurement properties (or a reference to a study on measurement properties) of the comparator instrument(s) in any study population | No information on the measurement properties of the comparator instrument(s), or evidence of insufficient measurement properties of the comparator instrument(s) |  | ***7 (line 6-18)***  ***10 (line 1-7)*** |
| 1. Perform the analysis in a sample with an appropriate number of patients (taking into account expected number of missing values) | ≥100 patients | | 50-99 patients | 30-49 patients | <30 patients |  | ***13 (line 1)*** |
| 1. Use an appropriate time schedule for assessments of the PROM of interest and comparison instruments | PROM and comparison instrument(s) will be administered at the same time | | PROM and comparison instrument(s) not administered at the same time, but assumable that patient will not change in the interim period | PROM and comparison instrument(s) will not be administered at the same time, but unclear if patients will changed | PROM and comparison instrument(s) will not be administered at the same time, and patients are expected to change |  | ***9 (line 25)*** |
|  |  | |  |  |  |  |  |
| ***Statistical methods*** |  | |  |  |  |  |  |
| 1. Use statistical methods that are appropriate for the hypotheses to be tested | Statistical methods will be appropriate | | Assumable that statistical methods will be appropriate | Statistical methods will not be optimal | Statistical methods will NOT be appropriate |  | ***10 (line 25-26)***  ***11 (line 1-3)*** |
| 1. Provide a clear description of how missing items will be handled | The way missing items will be handled is clearly described | |  | The way missing items will be handled is not clearly described |  |  | ***-*** |

**Responsiveness**

Responsiveness is considered to indicate longitudinal validity. When a ‘gold standard’ is available, the criterion approach Part A of this box can be used. When testing hypotheses about change scores of PROMs compared to other outcome measurement instruments, Part B can be used; for comparison of changes scores of PROMs between subgroups Part C can be used; and when testing hypotheses about expected change scores of PROMs before and after intervention, Part D can be used. It is of major importance to define hypotheses in advance when assessing responsiveness of a PROM, to enable the authors to draw unbiased conclusions after data collection and analyses.

| **D. Construct approach: (i.e. hypotheses testing: before and after intervention)** |  |  |  |  |  |  |  |
| --- | --- | --- | --- | --- | --- | --- | --- |
|  | **very good** | | **adequate** | **doubtful** | **inadequate** | **NA** | ***Reported***  ***on page #*** |
| ***Design requirements***   1. Formulate challenging hypotheses regarding expected changes before and after intervention a priori (i.e. before data collection) | Hypotheses formulated including the expected changes stated | |  | Hypotheses vague or not formulated but possible to deduce what was expected | Unclear what was expected |  | ***-*** |
| 1. Provide an adequate description of the intervention to allow replication, including how and when they will be administered | Adequate description of the intervention | |  | Poor description of the intervention | NO description of the intervention |  | ***7 (line 19-26)*** |
| 1. Use an appropriate time interval between first and second administration | Time interval appropriate | |  |  | Time interval NOT appropriate |  | ***10 (line 11-14)*** |
| 1. Describe anything likely to occur in the interim period (e.g. intervention, progressive disease, other relevant events) | Anything likely to occur during the interim period (e.g. treatment) is adequately described | |  | Unclear or NOT described what will likely to occur during the interim period |  |  | ***-*** |
| 1. Ensure that a proportion of the patients is likely to change (i.e. improvement or deterioration) on the construct to be measured | Part of the patients is likely to change (evidence provided) | | NO evidence provided, but assumable that part of the patients will change | Unclear if part of the patients will change | Patients will likely NOT change |  | ***-*** |
| 1. Perform the analysis in a sample with an appropriate number of patients (taking into account expected number of missing values) | ≥100 patients | | 50-99 patients | 30-49 patients | <30 patients |  | ***Table 5*** |
| **Statistical methods** |  | |  |  |  |  |  |
| 1. Ensure that the statistical methods are adequate for the hypotheses to be tested | Statistical methods are appropriate | | Assumable that statistical methods are appropriate | Statistical methods are not optimal | Statistical methods are NOT appropriate |  | ***11 (line 10-17)*** |
| 1. Provide a clear description of how missing items will be handled | The way missing items will be handled is clearly described | |  | The way missing items will be handled is not clearly described |  |  | ***-*** |
